# Fluctuating expression of miR-584 in primary and high-grade gastric cancer

**DOI:** 10.1186/s12885-020-07116-5

**Published:** 2020-07-07

**Authors:** Laleh Ebrahimi Ghahnavieh, Hossein Tabatabaeian, Zhaleh Ebrahimi Ghahnavieh, Mohammad Amin Honardoost, Mansoureh Azadeh, Mohamad Moazeni Bistgani, Kamran Ghaedi

**Affiliations:** 1 Department of Biology, Tehran-east Payame Noor University, Tehran, Iran; 2 Department of Cell and Molecular Biology and Microbiology, Faculty of Biological Science and Technology, University of Isfahan, Isfahan, Iran; 3 Anahid Cancer Clinic, Isfahan Healthcare City, Isfahan, Iran; 4 Department of Medical Education, Faculty of Medicine, Shahrekord University of Medical Sciences, Shahrekord, Iran; 5 Zist-Fanavari Novin Biotechnology Institute, Isfahan, Iran; 6 Department of Surgery, Faculty of Medicine, Shahrekord University of Medical Sciences, Shahrekord, Iran; 7 Department of Cellular Biotechnology, Cell Science Research Center, Royan Institute for Biotechnology, ACECR, Isfahan, Iran

**Keywords:** Gastric cancer, miR-584, Helicobacter pylori, Computational analysis

## Abstract

**Background:**

Gastric cancer is the fifth most common cancer worldwide. Along with environmental factors, such as *Helicobacter pylori* (*H. pylori*) infection, genetic changes play important roles in gastric tumor formations. miR-584 is a less well-characterized microRNA (miRNA), with apparent activity in human cancers. However, miR-584 expression pattern in gastric cancer development has remained unclear. This study aims to analyze the expression of miR-584 in gastric cancer samples and investigates the associations between this miRNA and *H. pylori* infection and clinical characteristics.

**Methods:**

The expression level of miR-584 was studied in primary gastric cancers versus healthy control gastric mucosa samples using the RT-qPCR method. The clinical data were analyzed statistically in terms of miR-584 expression*.* In silico studies were employed to study miR-584 more broadly in order to assess its expression and find new potential target genes.

**Results:**

Both experimental and in silico studies showed up-regulation of miR-584 in patients with gastric cancer. This up-regulation seems to be induced by *H. pylori* infection since the infected samples showed increased levels of miR-584 expression. Deeper analyses revealed that miR-584 undergoes a dramatic down-regulation in late stages, invasive and lymph node-metastatic gastric tumors. Bioinformatics studies demonstrated that miR-584 has a substantial role in cancer pathways and has the potential to target STAT1 transcripts. Consistent with the inverse correlation between TCGA RNA-seq data of miR-584 and STAT1 transcripts, the qPCR analysis showed a significant negative correlation between these two RNAs in a set of clinical samples.

**Conclusion:**

miR-584 undergoes up-regulation in the stage of primary tumor formation; however, becomes down-regulated upon the progression of gastric cancer. These findings suggest the potential of miR-584 as a diagnostic or prognostic biomarker in gastric cancer.

## Background

Gastric cancer, as the fifth most common malignancy, is the third cause of cancer-related death worldwide in both genders [1]. The pathogenesis of gastric cancer involves both genetics and environmental parameters [2]. A declining trend of gastric cancer incidence over the last decades obviously shows the significance of lifestyle, e.g. widespread food refrigeration rather than salt as the main form of preservation [3], and environmental factors, such as *Helicobacter pylori* (*H. pylori*) eradication. *H. pylori* infection is one of the noticeable correlated factors with gastric neoplasm through modulating inflammatory signaling pathways in the mucosa of the stomach. This infection can promote the risk of gastric cancer more than six-fold [4, 5].

Genetically, microRNAs (miRNAs) are important molecules that are deregulated in gastric cancer [6–9]. miRNAs are an emerging class of non-coding RNAs containing less than 24 nucleotides, which bind mainly to the 3′-UTR of their target mRNAs. These molecules play a role as post-transcriptional regulatory factors through either mRNA degradation or transcriptional repression of the targeted mRNAs [10]. miRNAs contribute to determining cell fate by means of regulating cellular processes such as proliferation and apoptosis [11]. Inevitably, dysregulation of miRNAs displays their association with various pathological effects, such as inflammatory process and precancerous lesions to malignancy progression [12, 13]. Notably, *H. pylori* infection is one of the vital factors responsible for miRNA dysregulation through regulating the inflammatory mediators [14–17]. miR-21 [18, 19], miR-25 [19], miR-93 [20], miR-146a [21], miR-155 [22], miR-196 [23], miR-200b/c [15, 24], miR-222 [25], miR-223 [20], miR-584 and miR-1290 [26] are the well-known *H. pylori*-induced up-regulated miRNAs, while let-7b [24, 27], miR-103 [24], miR-370 [28], miR-371, miR-372, miR-373 [29], miR-375 [24] and miR-449 [30] are the down-regulated ones.

More recently, miR-584 has been reported to be up-regulated in intestinal metaplasia and human primary colon cancer tissues, induced by the *H. pylori* infection. miR-584, in turn, promotes the epithelial-mesenchymal transition (EMT) via targeting FOXA1 gene [26]. Dysregulation of miR-584 has been also reported in gastric cancer [31]. However, there is a scientific gap regarding the expression of miR-584 in gastric cancer in the context of *H. pylori* infection. Therefore, the main aim of this study was to evaluate the expression level of miR-584 in normal gastric mucosa specimens along with malignant gastric mucosa tissue patients and analyze its association with *H. pylori* infection.

## Methods

### Patients and control samples

Tumor samples (*n* = 26) were obtained from the patients undergoing gastrectomy surgery at Al-Zahra Hospital, Isfahan, Iran, and Kashani Hospital, Shahre Kord, Iran. In addition, endoscopy samples (*n* = 11) were taken from healthy volunteers and used as control samples. Immediately after obtaining tissue samples, they were submerged in a 2 ml RNase-free containing RNAlater™ Stabilization Solution (Ambion, Austin, Texas) and transferred to a laboratory on ice for subsequent analyses. Before specimen collection, the stomach biopsies were taken to detect *H. pylori* infection using Rapid Urease Detection test (*Helicotec*UT Plus, Taiwan) in both patient and control groups. The normal/tumor status of the samples was verified by four expert pathologists. Ethically, the samples were collected in accordance with the guidelines issued by the Ethics Committee of Al-Zahra Hospital, regarding 1964 Helsinki declaration. Moreover, informed consent was gained from all studied individuals. The characteristics of the samples including age, sex and *H. pylori* infection are listed in Table 1.

**Table 1:** Patient sample characteristics

Variable		Control (***n*** = 11)	Case (***n*** = 26)	***p***
**Age (SD)**		49.73 (21.45)	61.13 (13.54)	0.055 *
**Sex**	**Woman**	4	6	0.406 **
	**Man**	7	20	
***H. pylori*infection**	**Negative**	5	12	0.969 **
	**Positive**	6	14	

*SD* Standard deviation, *H. pylori* Helicobacter pylori

* Independent t-test

** Chi-square test

### RNA extraction

Total RNA from tissue specimens was extracted using Hybrid-R™ miRNA kit (GeneAll, Seoul, South Korea). The quality of extracted RNAs determined according to 260/280 absorbance ratio, measured by the NanoDrop spectrometer (Thermo Scientific, USA). In order to remove potential DNA contamination, small RNA samples underwent RNA-free DNase (TaKaRa, Japan) treatment.

### cDNA synthesis and RT-qPCR

Normal cDNA synthesis was performed by RevertAid Reverse Transcriptase (Thermofisher) according to the manufacturer’s protocol. STAT1 primers were designed with Oligo Primer Analysis software (DBA Oligo, Inc.) as Forward: 5′-TGAACTTACCCAGAATGCCC-3′, and Reverse: 5′-CAGACTCTCCGCAACTATAGTG-3′.

cDNA synthesis for miR-584 and U6 (reference control) was achieved by using a Universal cDNA Synthesis Kit (Exiqon, Denmark), according to manufacturer’s instruction. Real-time quantitative PCR reactions were carried out in triplicate by using standard protocols with an ABI PRISM 7500 instrument (Applied Biosystems, USA). cDNA products were added to a master mix comprising 10 pmol/μl of each miR-584 and U6 primers (Exiqon, Denmark) and 2 U of SYBR premix ExTaq II (TaKaRa, Japan). Real-time data were evaluated and reported based on 2^-∆∆CT^ method to calculate the fold change in miR-584 expression among different groups. The PCR program conditions were: 95 °C for 5 min (min) followed by 40 cycles of 95 °C for 5 s (s), 60 °C for 20 s and 72 °C for 30 s.

### Statistical analysis

All statistical analyses were performed by SPSS statistical software, version 20.0 (SPSS Inc., Chicago, IL, USA). ANOVA test was used and followed by analyzing with a non-parametric Mann– Whitney post hoc test to evaluate and assess the potential of miR-584 as a diagnostic indicator of disease severity to differentiate between gastric malignancy and normal tissue with regards to *H. pylori* infection. Linear regression analysis was performed to analyze the relationship between stages and survival rates to miR-584 expression in human gastric cancers. Results were considered as statistically significant at *P* values equal or less than 0.05.

### In silico analyses

The TCGA data (https://www.cancer.gov/tcga) were analyzed to examine the expression levels of miR-584 in paired tumor and adjacent control samples of stomach adenocarcinoma patients. Besides, ENCORI (starBase) publicly available online tool [32] was used to analyze the differential expression of miR-584 in normal and tumor samples, and also to analyze the correlation between miR-584 and its host and predicted target genes. The KM Plotter database was used to investigate the survival rate of clinical patients in terms of the expression level of miR-584 [33].

In order to perform molecular enrichment analysis on miR-584 targetome and to determine the signaling pathways that are statistically involved, we used several online databases of miRWalk [34] and miRTarBase [35, 36] to achieve predicted and validated targets of miR-584 particularly. Next, using UniGene database (http://www.ncbi.nlm.nih.gov/unigene/) and subsequent EST profile, we investigated the filtered targetome expression in the stomach and gastrointestinal tumors. Finally, miR-584 targetome expressed in stomach and gastrointestinal tumor was assigned to the database for annotation, visualization and integrated discovery (DAVID) online bioinformatics database, version 6.7 [37] arranged with Kyoto Encyclopedia of Genes and Genomes (KEGG) pathway analysis [38] to recognize the most statistical correlated signaling pathways and molecular networks with miR-584 targetome.

## Results

### miR-584 is highly expressed in tumor samples

**Fig. 1:**
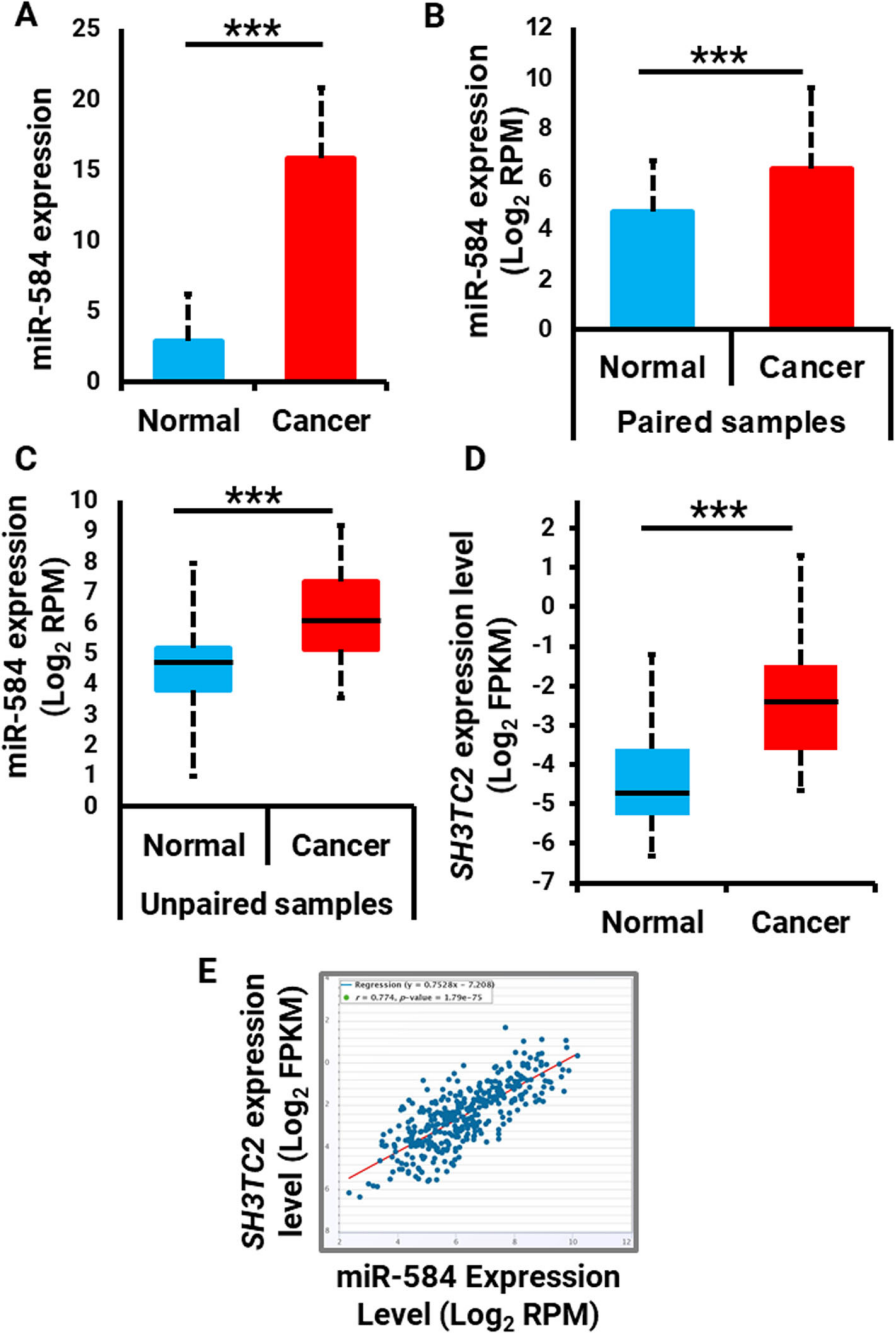
**a** Analysis of miR-584 expression in the patient’s tumor versus normal tissue. **b** In silico analysis of miR-584 in paired TCGA clinical gastric cancer and normal gastric mucosa samples. c In silico analysis of miR-584 in unpaired TCGA clinical gastric cancer samples. **d** Analysis of SH3TC2 expression in the patient’s tumor versus normal tissue. **e** Correlation between miR-584 and SH3TC2 expression gastric cancer samples. *** P < 0.005

The expression level of miR-584 was determined by RT-qPCR, followed by the statistical analysis in four groups, including patients with *H. pylori* infection (*n* = 14), patients without *H. pylori* infection (*n* = 12), control samples infected by *H. pylori* (*n* = 6) and non-infected control samples (*n* = 5). The expression pattern of miR-584 was normalized to U6. Primer specificity for miR-584 and U6 was evaluated by agarose gel electrophoresis (Supplementary Figure1). Our analyses revealed that miR-584 expression was approximately 5.3 times higher in gastric cancer cases as compared to the control samples (*P* < 0.001) (Fig. 1a). The differential expression of miR-584 was further studied using publicly available online tools of TCGA and ENCORI. Analyzing the TCGA data of stomach adenocarcinoma in the paired case-control samples significantly showed the upregulation of miR-584 (adjusted *P* < 0.001) (Fig. 1b). Consistently, the analysis of unpaired samples in ENCORI databased revealed significant and obvious higher expression of miR-584 in the tumor samples (adjusted *P* < 0.001) (Fig. 1c). Taken together, the in silico data strongly demonstrate the overexpression of miR-584 overexpression in gastric cancer samples, which has been validated in our study on the clinical specimens. The TCGA data were further studied to find whether miR-584 up-regulation is mediated by epigenetic alteration or amplification. First, we demonstrated that miR-584 is located in the intronic region of SH3TC2 gene. The transcription of these two genes might be regulated by the same promoter region as SH3TC2 was also shown to be up-regulated in gastric cancer (*P* = 1.7e-8) (Fig. 1d), which significantly and strongly correlated with miR-584 in gastric (*P* = 1.79e-75, *r* = 0.774) (Fig. 1e) and other panels of human cancers (Supplementary Figure 2). The TCGA gene copy number analysis revealed that SH3TC2 gene up-regulation is not mediated by gene amplification nor epigenetic alterations. These data collectively suggest that SH3TC2 gene, and thereby its intronic miR-584, are up-regulated in cancer probably in response to the higher expression/activity of their transcription factors. The ChIP-seq data obtained from the UCSC genome browser showed that SH3TC2/miR-584 locus is regulated by c-Jun and c-Fos transcription factors, which are known up-regulated oncoproteins in gastric cancer [39, 40].

### miR-584 expression is affected by *H. pylori* infection

**Fig. 2:**
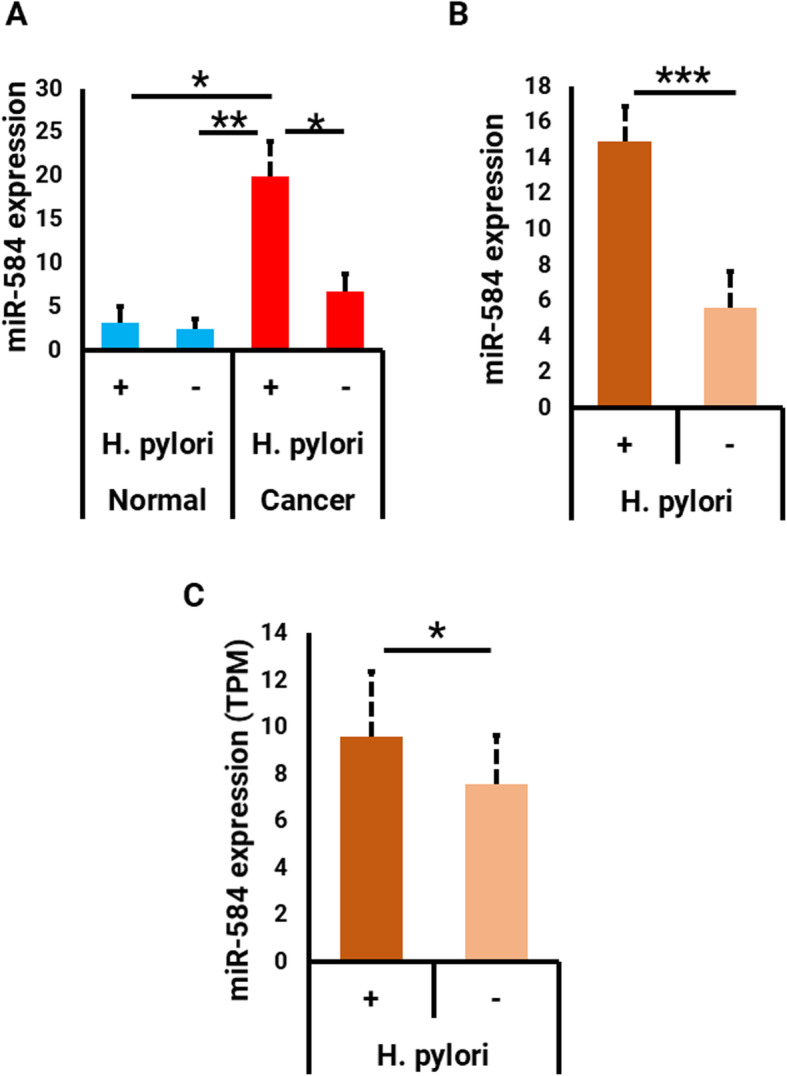
**a** Analysis of miR-584 expression in four *H. pylori*-categorized normal and tumor samples. **b** Analysis of miR-584 expression in *H. pylori*-positive and negative samples. **c** Analysis of miR-584 expression in *H. pylori*-categorized TCGA clinical samples. * *P* < 0.05, ** P < 0.01, *** P < 0.005

Dividing the samples in terms of *H. pylori* infection, the data significantly showed a statistically elevated expression of miR-584 in the *H. pylori*-infected patients in comparison with other groups including *H. pylori*-infected controls (*P* = 0.018), non-infected controls (*P* = 0.007) and *H. pylori*-negative patients (*P* = 0.011). However, there was no significant differences between the miR-584 expression in *H. pylori*-negative patients and *H. pylori*-infected controls (*P* = 0.609) or non-infected controls (*P* = 0.054) (Fig. 2a). Comparing the control samples, although the expression of miR-584 increased slightly in the *H. pylori*-infected samples, the difference was not significant (*P* = 0.928) (Fig. 2a). Furthermore and independent of tumor/control status, miR-584 was expressed significantly higher in *H. pylori*-infected samples as compared to non-infected ones, with approximately 2.6 fold change increase (*P* = 0.005) (Fig. 2b). To examine this hypothesis in another cohort, the TCGA samples, that had been recently tested for *H. pylori* infection using the methods described in the Zhang et al. study [41], were then analyzed in terms of miR-584 expression. Likewise, this analysis displayed a significant correlation between *H. pylori* infection and miR-584 expression (*P* = 0.034) (Fig. 2c). Collectively, the findings suggest that the elevated expression of miR-584 could be driven by *H. pylori* infection in gastric cancer. It remains to be validated whether *H. pylori* infection can up-regulate miR-584 in gastric cancer based on in vitro assays.

### miR-584 does not correlate with the patients’ demographics

We then asked whether the expression of miR-584 correlates with the age, blood group or the sex of the studied patients. Our analyses showed no significant correlation or association between either age (*P* = 0.309), sex (*P* = 0.745) or blood groups (*P* = 0.774). Next, we interrogated whether miR-584 is associated with the clinical features of gastric cancer patients.

### Association of miR-584 expression level and clinicopathological factors

To delineate the clinical significance of miR-584, we analyzed the correlation between miR-584 levels and the patient’s clinicopathological factors in greater detail. To study the potential correlation with gastric cancer stages, all tumor samples were reviewed by the pathologist after Hematoxylin & Eosin (H&E) staining and were categorized into different clinical stages (I-IV) based on AJCC/UICC Tumor Node Metastasis (TNM) staging system.

**Fig. 3:**
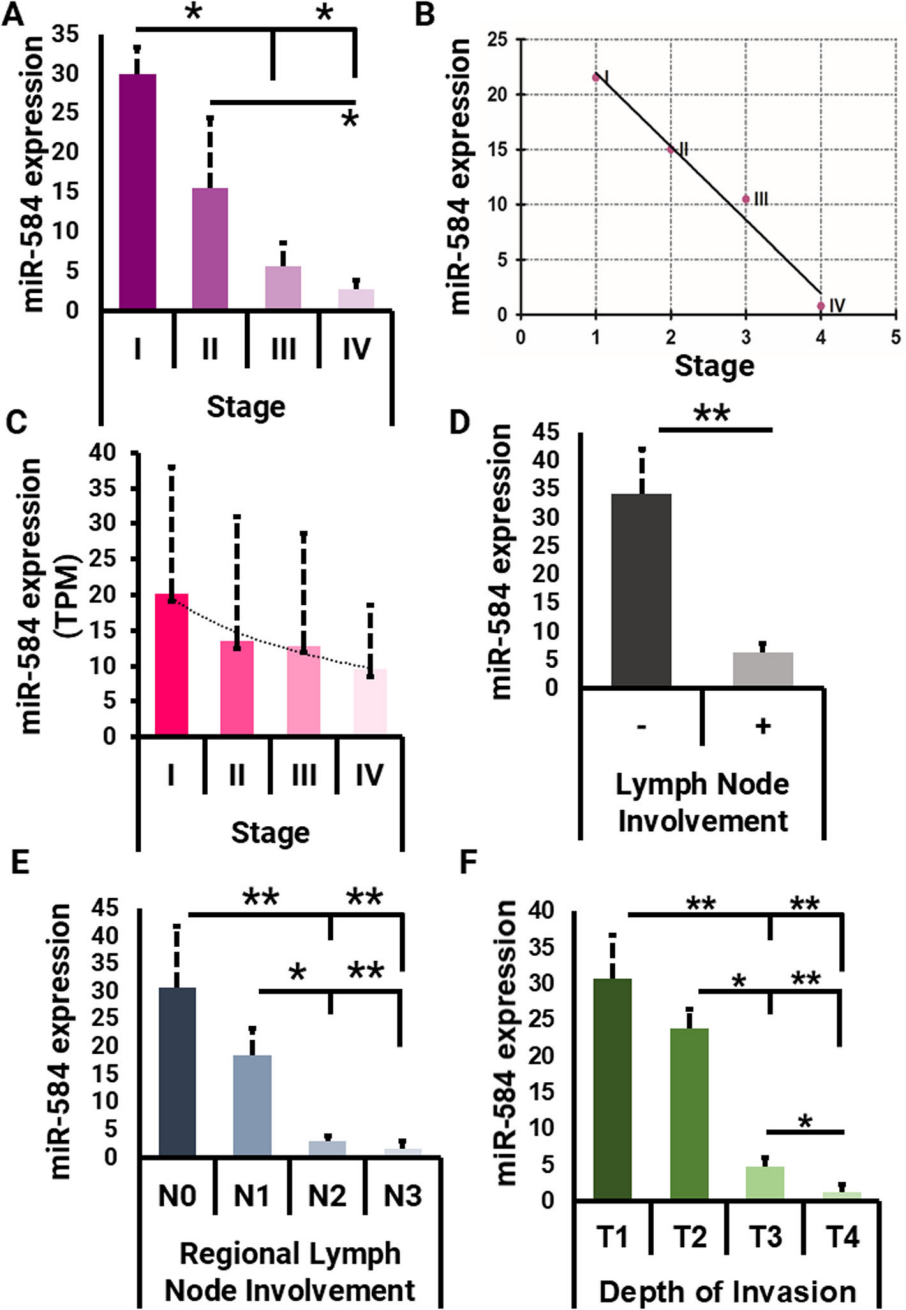
**a** Analysis of miR-584 in different gastric cancer stages. **b** Correlation of miR-584 and different stages of gastric cancer samples. **c** Analysis of miR-584 expression in different gastric cancer stages of TCGA gastric cancer samples. **d** Analysis of miR-584 in lymph node-negative and positive gastric tumors. **e** Analysis of miR-584 among tumors with different regional lymph node involvement. **f** Analysis of miR-584 expression among gastric tumors with different depths of invasion. * *P* < 0.05, ** P < 0.01

Interestingly, there was a significant difference between the expression level of miR-584 in different staging groups, including stage I and III (*P* = 0.048), I and IV (*P* = 0.030), and II and IV (*P* = 0.038) (Fig. 3a). Furthermore, linear regression analyses showed that miR-584 expression was negatively correlated with gastric malignancy stages, in a way that the expression level of this miRNA was higher in the early stages of gastric cancer and it decreased in the progression of malignancy (*P* < 0.001, Spearman test) (Fig. 3b). The high-throughput analysis of TCGA data also revealed a similar trend, i.e. elevated miR-584 expression in early and lower expression in the higher stages (Fig. 3c). These results suggest that this miRNA could be specifically a cancer-promoting factor which undergoes down-regulation during cancer progression. In line with this finding, higher expression of miR-584 was shown to be associated significantly with the absence of regional lymph node metastases, in comparison with lymph node metastatic samples (*P* = 0.005) (Fig. 3c). Interestingly, we did find a significant difference of miR-584 expression in N0 level, as compared to N2 (*P* = 0.001) and N3 (*P* = 0.001). Additionally, the expression of miR-584 in N1 groups was different from N2 (*P* = 0.028) and N3 (*P* = 0.002). Nevertheless, there were no significant differences between N0 and N1 (*P* = 0.306) or N2 and N3 (*P* = 0.129) (Fig. 3d).

Moreover, evaluation of the miR-584 expression and the degree of invasion to the gastric wall showed a significant difference in T1 vs. T3 (*P* = 0.004) and T4 (*P* = 0.009); but not to T2 (*P* = 0.931). Furthermore, a significant different expression of miR-584 was observed when comparing T2 to T3 (*P* = 0.021) and T4 (*P* = 0.003), and T3 to T4 (*P* = 0.008). (Fig. 3e). Collectively, the miR-584 expression pattern reveals that this small non-coding RNA is highly expressed upon the formation of gastric tumors. However, its regulation declines significantly with the progression of cancer.

**Fig. 4:**
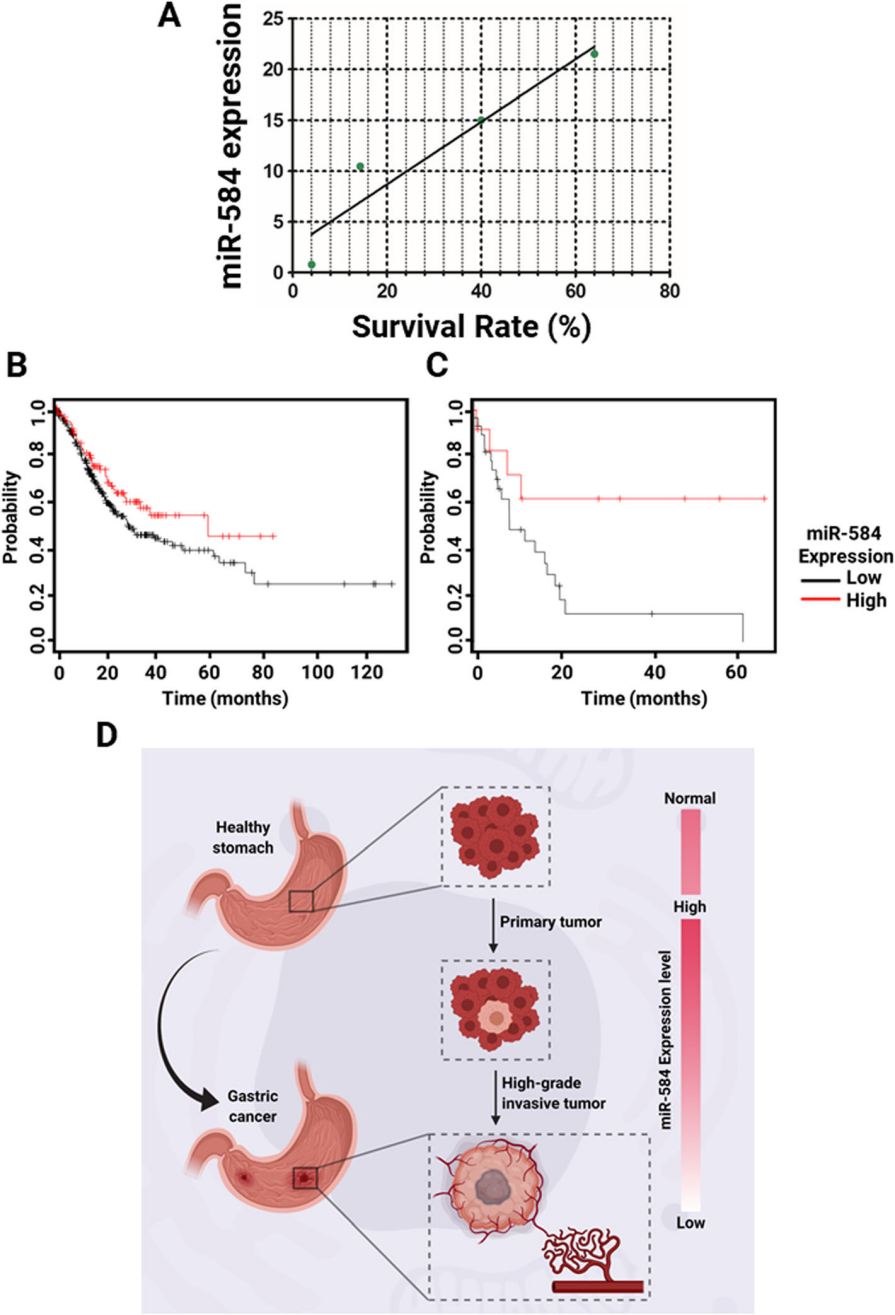
**a** Survival analysis of miR-584 expression in four different gastric tumor stages. **b** In silico survival analysis of miR-584 expression in clinical gastric cancer subjects. **c** In silico survival analysis of miR-584 expression in patients with stage IV of gastric cancer. **d** Proposed schematic of miR-584 expression pattern in gastric cancer development

These provocative findings prompted us to check whether miR-584 correlates with the higher survival rate of the patients. Linear regression analysis revealed clear segregation of survival rates between the four staging groups (Fig. 4a). The regression curve demonstrates a strong correlation between miR-584 level and 5-year survival rate of the patients. To assess this finding in a larger population, KM Plotter online tool was used. Analyzing the survival rate of 431 stomach adenocarcinoma patients clearly showed that higher expression of miR-584 is correlated with lower overall survival of the patients (Log rank *P* = 0.05, hazard ratio = 0.72, 95% CI: 0.50–1.03) (Fig. 4b). Further analyses revealed that patients with a higher expression of miR-584 survived for median of 56.2 months, while the patients with a low expression only survived for a median of 26.7 months. More interestingly, studying the patients with stage IV of adenocarcinoma showed a greater effect of miR-584 expression (Log rank *P* = 0.032, hazard ratio = 0.32, 95% CI: 0.11–1.96) (Fig. 4c). Hypothetically, although miR-584 is dramatically up-regulated in primary gastric tumors, it undergoes down-regulation upon the progression of cancer, contributing to the higher stages and lower survival of the patients. The schematic proposed role of miR-584 in gastric cancer initiation and progression is shown in Fig. 4d.

### Computational analysis of miR-584 targetome proposed a possible inducing role of miR-584 in cancer

**Fig. 5:**
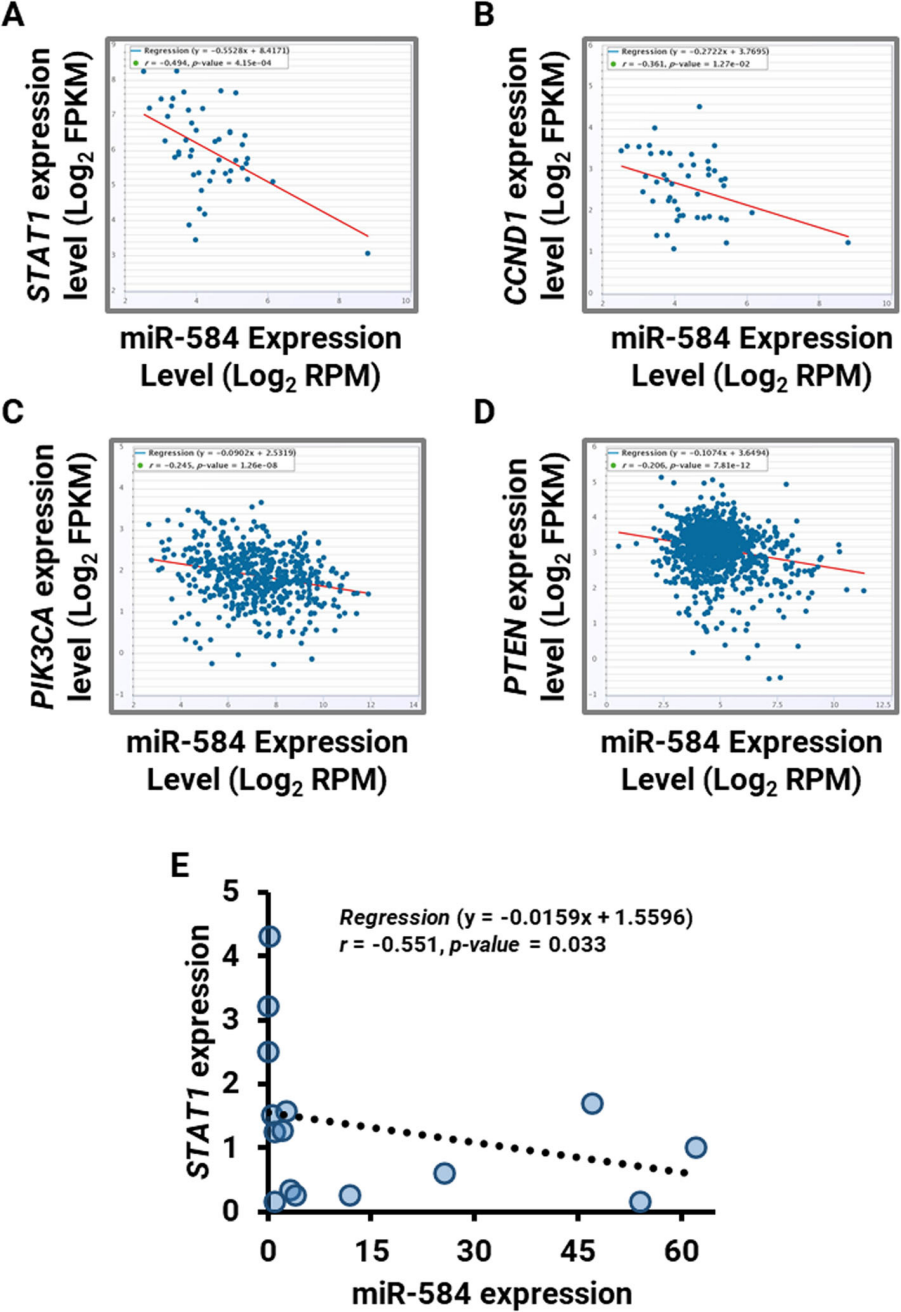
In silico correlation of miR-584 and **(a)** STAT1, **(b)** CCND1, **(c)** PIK3CA and **(d)** PTEN expression levels in TCGA samples. **e** Correlation of miR-584 and STAT1 expression levels in 5 normal gastric mocusa and 10 gastric cancer clinical samples

To help define how miR-584 may be related to gastric cancer, molecular signaling pathway enrichment analysis as already described in the materials and methods was performed. Using miRTarBase and miRwalk databases, 112 and 1552 mRNAs were specified as validated and predicted targets of miR-584, respectively (data not shown). Among the list, 65 validated and 670 predicted mRNA targets were reported to be expressed in stomach and gastrointestinal tumors, achieved from the UniGene database. These target genes were selected as miR-584 targetome for further molecular enrichment analysis. Imputing Entrez IDs as official gene symbols of selected miR-584 targetome into the functional annotation tool of DAVID determined the statistically significant association of imputed genes with several KEGG signaling pathways. The set of imputed genes representing the miR-584 targetome was chiefly associated with several KEGG signaling pathways showing that target genes were significantly involved in cancer pathways and other oncogenic/tumor suppressor transduction systems including Wnt signaling, TGF-β signaling, adherence junction and VEGF signaling pathways (Table 2). The enriched KEGG pathways in cancer are shown in Supplementary Figure 3. Interestingly, among these targets, EST1 and MAPK1 are validated targets of miR-584 as reported by experimental technology evidence. STAT1, PTEN, CCND1 and PIK3CA genes were among the top-score unvalidated miR-584 targetome. In silico correlation analysis performed via ENCORI database revealed that there were significant inverse correlations between miR-584 and these transcripts (Fig. 5a-d). STAT1 was selected for correlation analysis in 10 gastric cancer and 5 normal gastric mucosa in-house clinical samples due to its highest r value. Interestingly, qPCR analysis and Spearman’s correlation test showed a significant negative correlation between miR-584 and STAT1 transcript expression (*r* = − 0.551, *P* = 0.033) (Fig. 5e). Taken together, miR-584 may have an unvalidated cancer-related targetome that includes PTEN, CCND1, PIK3CA and STAT1. The latter showed a negative correlation in both TCGA and in-house clinical samples. The end-point in vitro validation using luciferase reported assay remains to be performed to validate the predicted targetome of miR-584.

**Table 2:** Top statistically related signaling pathways to miR-584 targetome obtained from KEGG database through DAVID tool

Rank	KEGG Pathway	Number of genes (%)	P*
1	Pathways in cancer	21 (2.9)	5.4E­2
2	Regulation of actin cytoskeleton	19 (2.6)	3.4E­3
3	Focal adhesion	17 (2.4)	9.1E­3
4	Ubiquitin mediated proteolysis	15 (2.1)	1.6E­3
5	Chemokine signaling pathway	14 (1.9)	4.7E­2
6	Endocytosis	13 (1.8)	8.1E­2
7	Fc gamma R­mediated phagocytosis	12 ()	1.8E­3
8	Chronic myeloid leukemia	11 (1.7)	9.9E­4
9	Insulin signaling pathway	11 (1.5)	5.4E­2
10	Wnt signaling pathway	11 (1.5)	9.8E­2
11	Pancreatic cancer	10 (1.4)	2.8E­3
12	Adherens junction	10 (1.4)	4.4E­3
13	Vascular smooth muscle contraction	9 (1.2)	9.4E­2
14	Colorectal cancer	9 (1.2)	2.3E­2
15	TGF­beta signaling pathway	9 (1.2)	2.8E­2
16	Oocyte meiosis	9 (1.2)	8.7E­2
17	Long­term potentiation	8 (1.1)	2.2E­2
18	Phosphatidylinositol signaling system	8 (1.1)	3.4E­2
19	Endometrial cancer	7 (1)	2.0E­2
20	Acute myeloid leukemia	7 (1)	3.3E­2
21	Melanoma	7 (1)	7.4E­2
22	B cell receptor signaling pathway	7 (1)	9.1E­2
23	VEGF signaling pathway	7 (1)	9.1E­2
24	Non­small cell lung cancer	6 (0.8)	7.3E­2
25	Dorso­ventral axis formation	4 (0.6)	8.4E­2

* The threshold of EASE score, a modified Fisher Exact *P*-value, for gene-enrichment analysis

## Discussion

There are emerging reports pertaining to the pathogenesis capabilities of miRNAs in cancer. Such studies have demonstrated the great importance of miRNAs as diagnostic/prognostic biomarkers with therapeutic potential. Among several miRNAs, a number of studies reported the association between miR-584 with different cancers such as lung [42], breast [43] and neuroblastoma [44]. Consistent with these findings, miR-584 may contribute to other malignancies such as gastric cancer.

In an effort to elucidate the role of miR-584 in gastric cancer - for the first time and to the best of our knowledge - we performed a case-control study and evaluated miR-584 expression level in 26 cases and 11 controls of the non-neoplastic gastric mucosa. Furthermore, in silico analyses obtained by the publicly available online tools were employed to assess the key findings.

Analysis of the expression of miR-584 in gastric tumor and healthy stomach tissues showed that miR-584 is significantly up-regulated in the primary gastric cancer tissues. The in silico studies on the paired and unpaired samples significantly displayed the miR-584 expression in gastric cancer patients. We further analyzed the in-house and TCGA data and showed that the up-regulation of miR-584 could be mediated by *H. pylori* infection since the infected samples showed higher expression of miR-584 significantly. Interestingly, the known *H. pylori*-induced transcription factors, c-Jun and c-Fos [45, 46], were shown to be the potential miR-584 transcription drivers. Collectively, we propose *H. pylori* ➔ c-Jun/c-Fos ➔ miR-584 route as a proposed mechanistic route of miR-584 up-regulation in gastric cancer. This hypothesis remained to be further studied and validated by in vitro biochemical experiments.

Despite the miR-584 expression up-regulation in gastric cancer patients, the analyses of low versus high-grade tumor samples clearly showed that the expression of miR-584 undergoes down-regulation upon the progression of gastric cancer. miR-584 has indeed lower expression in higher stages, invasive and lymph node metastatic samples. Interestingly, the bioinformatics studies on the survival rate of gastric cancer subjects reveal that the patients with higher expression of miR-584 survived for a longer time. These findings suggest that miR-584 overexpression might be required for gastric tumorigenesis, while it has to be down-regulated upon tumor progression. Further in vitro biochemical studies are highly recommended through which the biological functions such as cell growth and invasion of gastric cancer cell lines can be examined upon miR-584 overexpression and downregulation. Narasimhan et al. reported the up-regulation of miR-584 in colorectal cancer. Interestingly, the higher expression of this miRNA was only observed in colon adenoma and not carcinoma. In line with our findings, this study supports that miR-584 overexpression is only required for the primary levels of tumorigenesis [47].

As discussed before, we showed that there is a significantly higher miR-584 expression in *H. pylori*-infected samples, especially in tumors. Consistent with our finding, Zhu et al. demonstrated the NFκB-mediated up-regulation of miR-584 in response to *H. pylori* infection, which in turn resulted in the induction of intestinal metaplasia of gastric epithelial cells [26]. Furthermore, miR-584 up-regulation could also be driven in Porphyromonas gingivalis infection and inflammatory diseases [48, 49]. These findings strongly indicate that miR-584 could be an important downstream target of inflammatory and pathogenic stimuli, contributing to stomach tumorigenesis. Performing in-situ hybridization of miR-584 in the sections including proper gastric gland, intestinal metaplasia and gastric cancer tissue can further prove this finding.

Controversially, Li et al. reported that miR-584 plays a role as a tumor suppressor miRNA in gastric cancer. In this study, the down-regulation of miR-584 was shown and the biochemical in vitro studies revealed that miR-584 inhibits the proliferation rate via targeting WW domain-containing E3 ubiquitin-protein ligase 1 (WWP1), which triggers the TGF-β signaling pathway [31]. However, they did not clearly specify the stage/grade, and more importantly, the status of *H. pylori* infection of the studied samples.

Although some miR-584 target transcripts have been validated, such as WWP1 and ROCK1 [50], many other potential targets are yet to be studied. Our in silico investigations have introduced a new subset of genes that can be potentially targeted by miR-584. Among them, STAT1, PTEN, PI_3_K and cyclin D1 are the most interesting targets. Our in silico analysis showed a significant negative correlation between miR-584 and either of the genes. The negative correlation of miR-584 and STAT1 was further validated by analyzing 15 samples using qPCR method. These genes have remained to be validated by biochemical in vitro studies in the future. This can be achieved by cloning the 3’UTR of the genes downstream of luciferase and investigate the luciferase activity upon overexpression or knockdown of miR-584.

## Conclusion

miR-584 has a fluctuating expression level in a normal stomach, primary tumors and progressed gastric cancer. This miRNA undergoes up-regulation in the early stages of primary tumor formation; however, it becomes down-regulated upon the progression of gastric cancer. These findings propose the potential of miR-584 as a diagnostic biomarker for early detection of gastric cancer and a prognostic factor to predict the clinical outcomes of the patients. The former can be studied by analyzing the miR-584 in the serum or saliva of gastric cancer and healthy individuals to determine whether this dysregulated molecule could serve as a diagnostic circulating miRNA in high-risk people.

## Supplementary information

**Additional file 1 Supplementary Figure 1.** Analysis of RT-qPCR products of miR-584 and U6 primers separated by agarose gel electrophoresis.

**Additional file 2 Supplementary Figure 2.** Correlation between miR-584 and SH3TC2 expression in a panel of human cancers including (A) brain lower grade glioma, (B) head and neck squamous cell carcinoma, (C) lung squamous cell carcinoma, (D) kidney renal papillary cell carcinoma, (E) skin cutaneous melanoma and (F) lung adenocarcinoma.

**Additional file 3 Supplementary Figure 3.** miR-584 targetome is involved in several important pathways including cancer pathways as well as Wnt signaling, TGF-beta signaling, adherence junction and VEGF signaling pathways, ranked as top related signaling pathways. The diagram collected from KEGG pathway. The unvalidated potential targets of miR-584 are marked by red asterisks.

## Data Availability

The data analyzed in this study will not be shared due to the policy of the Ethics Committee.

## Abbreviations

ChIP: Chromatin Immunoprecipitation
DAVID: Database for Annotation, Visualization and Integrated Discovery
EMT: Epithelial-mesenchymal Transition
ENCORI: The Encyclopedia of RNA Interactomes
FOXA1: Forkhead Box A1
*Helicobacter pylori*: *H. pylori*
H&E staining: Hematoxylin & Eosin staining
KEGG: Kyoto Encyclopedia of Genes and Genomes
KM: Kaplan Meier
MAP K1: Mitogen-Activated Protein Kinase 1
microRNAs: MiRNAs
NFκB: Nuclear Factor Kappa B Subunit
PI3K: Phosphatidylinositol-4,5-Bisphosphate 3-Kinase
ROCK1: Rho Associated Coiled-Coil Containing Protein Kinase 1
RT-qPCR: Reverse Transcriptase quantitative PCR
SH3TC2: SH3 Domain and Tetratricopeptide Repeats 2
STAT1: Signal Transducer and Activator of Transcription 1
TCGA: The Cancer Genome Atlas
TGF-β: Ransforming Growth Factor Beta
TNM: Tumor Node Metastasis
VEGF: Vascular Endothelial Growth Factor
WWP1: WW domain-containing E3 ubiquitin-protein ligase 1.
