# Supplementary figures and images for: Fluctuating expression of miR-584 in primary and high-grade gastric cancer
Source: BMC Cancer. 2020 Jul 7;20:621. doi: 10.1186/s12885-020-07116-5 (PMC7345521; doi:10.1186/s12885-020-07116-5)

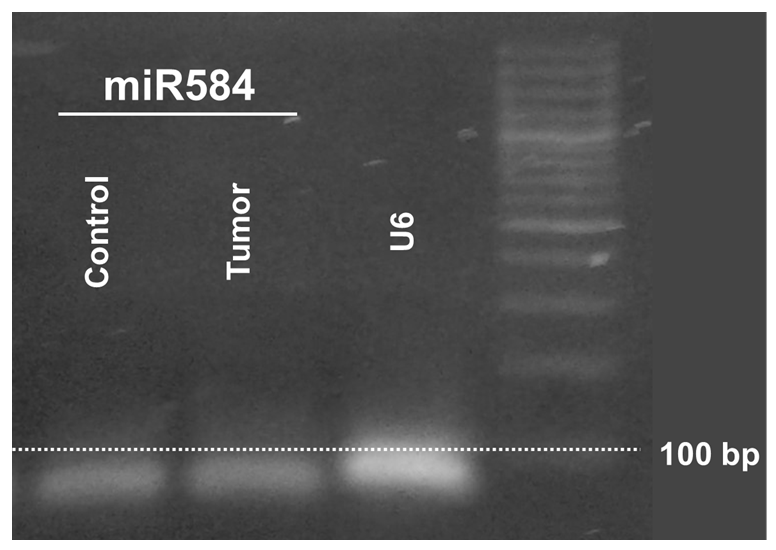

Supplement: Supplementary file 1 — Additional file 1 Supplementary Figure 1. Analysis of RT-qPCR products of miR-584 and U6 primers separated by agarose gel electrophoresis. [file 12885_2020_7116_MOESM1_ESM.tif]

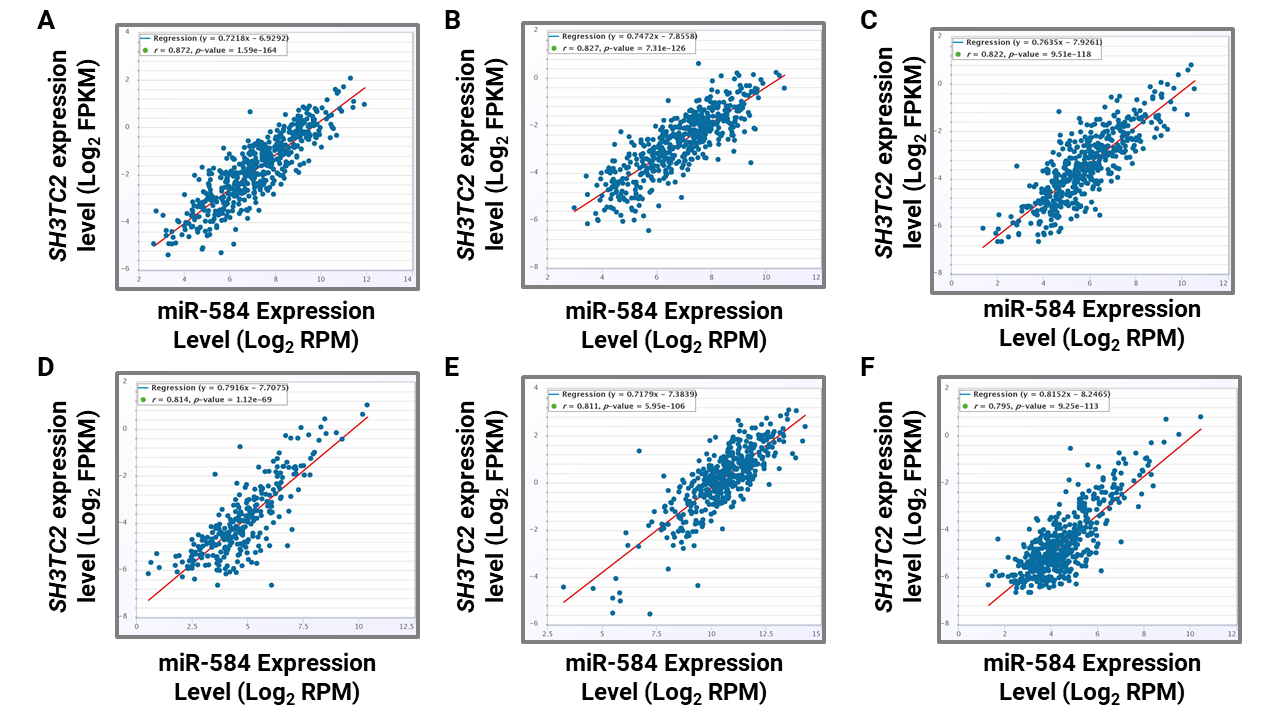

Supplement: Supplementary file 2 — Additional file 2 Supplementary Figure 2. Correlation between miR-584 and SH3TC2 expression in a panel of human cancers including (A) brain lower grade glioma, (B) head and neck squamous cell carcinoma, (C) lung squamous cell carcinoma, (D) kidney renal papillary cell carcinoma, (E) skin cutaneous melanoma and (F) lung adenocarcinoma. [file 12885_2020_7116_MOESM2_ESM.tif]

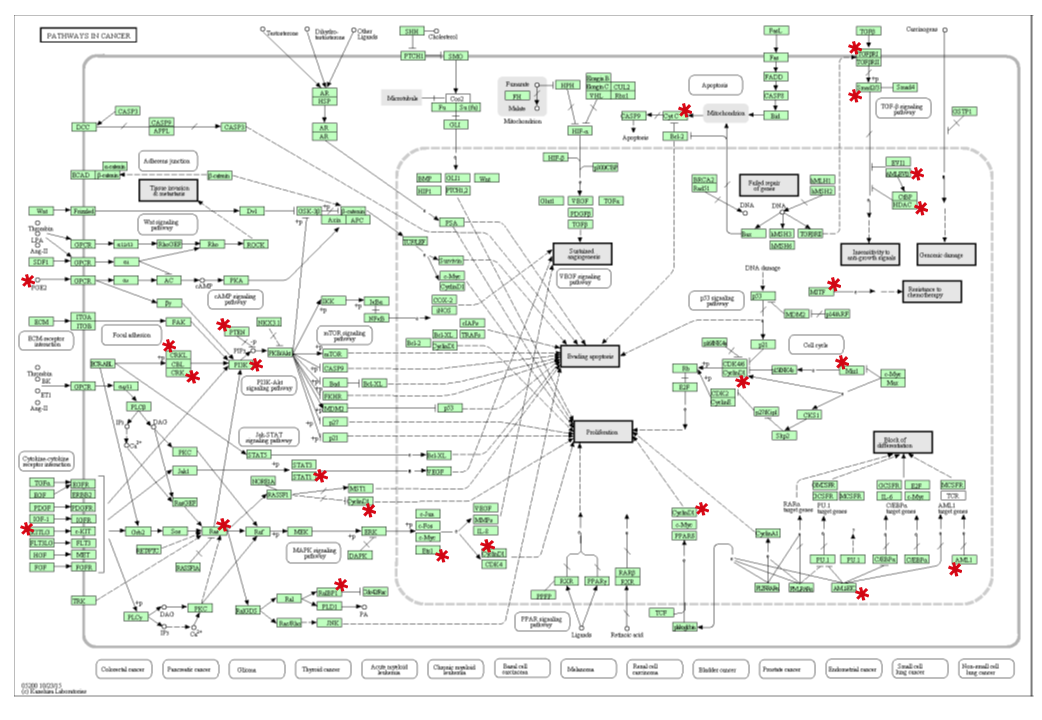

Supplement: Supplementary file 3 — Additional file 3 Supplementary Figure 3. miR-584 targetome is involved in several important pathways including cancer pathways as well as Wnt signaling, TGF-beta signaling, adherence junction and VEGF signaling pathways, ranked as top related signaling pathways. The diagram collected from KEGG pathway. The unvalidated potential targets of miR-584 are marked by red asterisks. [file 12885_2020_7116_MOESM3_ESM.tif]
